# Tunnel engineering to accelerate product release for better biomass-degrading abilities in lignocellulolytic enzymes

**DOI:** 10.1186/s13068-019-1616-3

**Published:** 2019-11-23

**Authors:** Zhenghui Lu, Xinzhi Li, Rui Zhang, Li Yi, Yanhe Ma, Guimin Zhang

**Affiliations:** 1 State Key Laboratory of Biocatalysis and Enzyme Engineering, Hubei Collaborative Innovation Center for Green Transformation of Bio-resources, Hubei Key Laboratory of Industrial Biotechnology, School of Life Sciences, Hubei University, Wuhan, 430062 Hubei China; 2 Tianjin Institutes of Industrial Biotechnology, Chinese Academy of Science, Tianjin, 300308 China

**Keywords:** Xylanase, Buried active site, Tunnel-like structure, Lignocellulose biodegradation, Specific activity

## Abstract

**Background:**

For enzymes with buried active sites, transporting substrates/products ligands between active sites and bulk solvent via access tunnels is a key step in the catalytic cycle of these enzymes. Thus, tunnel engineering is becoming a powerful strategy to refine the catalytic properties of these enzymes. The tunnel-like structures have been described in enzymes catalyzing bulky substrates like glycosyl hydrolases, while it is still uncertain whether these structures involved in ligands exchange. Till so far, no studies have been reported on the application of tunnel engineering strategy for optimizing properties of enzymes catalyzing biopolymers.

**Results:**

In this study, xylanase S7-xyl (PDB: 2UWF) with a deep active cleft was chosen as a study model to evaluate the functionalities of tunnel-like structures on the properties of biopolymer-degrading enzymes. Three tunnel-like structures in S7-xyl were identified and simultaneously reshaped through multi-sites saturated mutagenesis; the most advantageous mutant 254RL1 (V207N/Q238S/W241R) exhibited 340% increase in specific activity compared to S7-xyl. Deconvolution analysis revealed that all three mutations contributed synergistically to the improved activity of 254RL1. Enzymatic characterization showed that larger end products were released in 254RL1, while substrate binding and structural stability were not changed. Dissection of the structural alterations revealed that both the tun_1 and tun_2 in 254RL1 have larger bottleneck radius and shorter length than those of S7-xyl, suggesting that these tunnel-like structures may function as products transportation pathways. Attributed to the improved catalytic efficiency, 254RL1 represents a superior accessory enzyme to enhance the hydrolysis efficiency of cellulase towards different pretreated lignocellulose materials. In addition, tunnel engineering strategy was also successfully applied to improve the catalytic activities of three other xylanases including xylanase NG27-xyl from *Bacillus* sp. strain NG-27, TSAA1-xyl from *Geobacillus* sp. TSAA1 and N165-xyl from *Bacillus* sp. N16-5, with 80%, 20% and 170% increase in specific activity, respectively.

**Conclusions:**

This study represents a pilot study of engineering and functional verification of tunnel-like structures in enzymes catalyzing biopolymer. The specific activities of four xylanases with buried active sites were successfully improved by tunnel engineering. It is highly likely that tunnel reshaping can be used to engineer better biomass-degrading abilities in other lignocellulolytic enzymes with buried active sites.

## Background

To address global energy demands and environmental concerns, continuous efforts have been made to produce liquid fuels on sustainable resources. Lignocellulosic materials are the most abundant renewable biomass in nature, promising to provide sufficient resources for substitution of fossil-derived fuels and chemicals [1]. Cellulose, hemicellulose and lignin are the main components in lignocellulosic materials; the intra- and inter-covalent bonds between these components are highly recalcitrant to enzymatic hydrolysis, being a bottleneck on the efficient bioconversion of cellulose into fermentable sugars [2]. Though preparing enzyme cocktails, including cellulase, xylanase, and pectinase, promise to improve the bioconversion efficiency of lignocellulose [3, 4], the high-cost enzymes impose obstacles for commercial practices [5]. Therefore, engineering lignocellulolytic enzymes with better catalytic properties to reduce high enzyme loadings is in urgent need for the next generation of biorefinery [6, 7].

Recently, we have utilized both directed evolution and rational protein design to engineer various kinds of carbohydrate hydrolases like cellulase, amylase and pectate lyase [8–10]. What we learned is semi-rational approach of constructing small but smart libraries could significantly increase the biocatalyst optimization efficiency [11–13]. Of which, the prerequisite is the identification of structural features governing certain enzymatic characteristics.

Many enzymes have their active sites buried and evolved access tunnels to aid ligands transportation between the active sites and surrounding solvent [14, 15]. Recent studies have demonstrated that the physicochemical properties of access tunnels have a significant influence on enzyme’s activity, stability and substrate specificity [14, 16, 17]. Therefore, tunnel engineering is becoming a relatively new strategy to optimize enzymes with buried active sites, especially for these acting on small substrates. Though tunnel-like structures have been observed in glycosyl hydrolases [18–20], whether these structures could be defined as access tunnels is still controversial, and little is known about their functionalities. Thus, there is no report of using tunnel engineering strategy to optimize enzymes catalyzing biopolymers.

Xylanase S7-xyl from *Bacillus halodurans* S7 (hereinafter referred to as S7-xyl) belongs to glycoside hydrolase family 10 (GH10), which exhibits broad temperature (more than 90% of activity was remained after 4-h incubation at 60 °C) and pH adaptabilities (its activity remains constant at pH from 5.5 to 10.5) [21]. Structurally, S7-xyl (PDB: 2UWF) has a 16-amino acid protrusion between helix α3a and α3c, resulting in a much deeper active cleft than many other xylanases [22]. Using the CAVER algorithm [23], we found that there were three tunnel-like structures in xylanase S7-xyl. Hence, S7-xyl is a good model to explore the functionalities of tunnel-like structures in biopolymer degrading enzymes.

In these study, structure-guided multi-sites saturation mutagenesis was performed to simultaneously reshape tunnel-like structures in S7-xyl, and structure–function relationships of these tunnels were analyzed structurally and kinetically. In addition, tunnel engineering strategy was applied to enhance the specific activities of three other xylanases sharing different sequence identity with S7-xyl. Namely, NG27-xyl was derived from *Bacillus* sp. strain NG-27, which has the highest activity at 70 °C and pH 8.4 with a half-life of 75 min at 70 °C and 70% of relative activity at pH 11 [24]. TSAA1-xyl from *Geobacillus* sp. TSAA1 has the highest activity at 80 °C and pH 8.5, maintaining high stability over a broad range of pH (6–12) and temperature (40–100 °C) [25]. N165-xyl was derived from *Bacillus* sp. N16-5 with a specific activity of 92.5 U/mg, which has optimal reaction conditions of 70 °C and pH 7 [26]. These results suggested that engineering the tunnel-like structures would be a novel strategy to optimize the catalytic performance of lignocellulolytic enzymes.

## Results

### Tunnel identification and engineering in xylanase S7-xyl

**Fig. 1:**
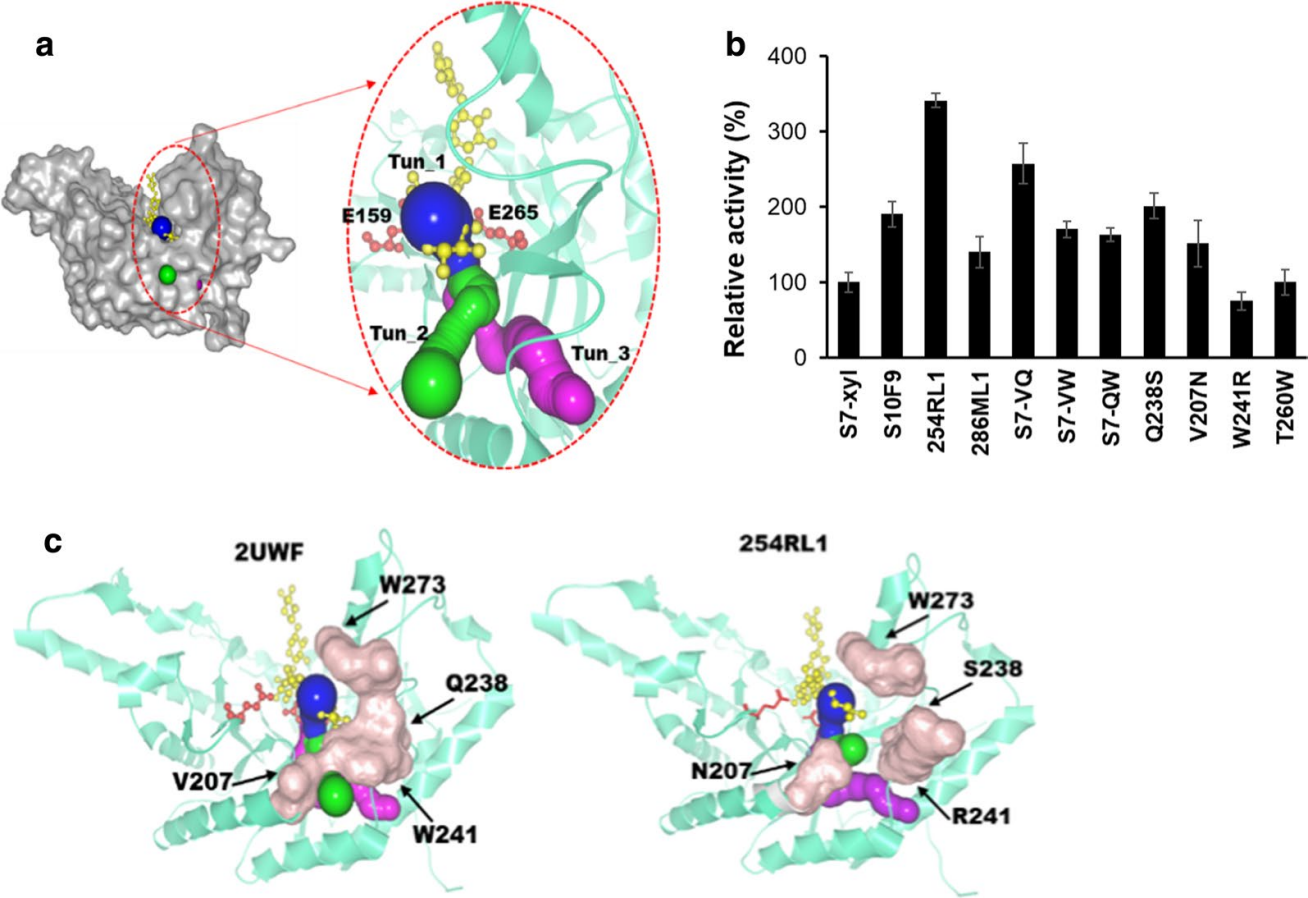
Structure analysis and activity measurement of xylanases S7-xyl and its mutants. **a** Molecular docking analysis was performed by AutoDock software in YASARA using hydrolyzed xylopentaose as ligand. The binding energy in the final docked structure was 9.49 kcal/mol. The nearest distance between ligand and the catalytic residues E159 and E265 are 2.2 Å and 3.5 Å, respectively. The overall structure of 2UWF is shown as gray surface. The ligand is shown as ball-and-stick model in yellow. Red sticks indicate the catalytic residues. The tunnel-like structures were calculated by CAVER and named as tun_1 (blue), tun_2 (green) and tun_3 (purple). **b** The relative activities of wild-type and the mutants. **c** Mapping the mutated residues on the structure of wild-type xylanase S7-xyl (2UWF) and the mutant 254RL1, respectively. Structures are shown as teal cartoon. The mutated residues are shown as pink surface

Enzymes with buried active sites usually evolved access tunnels to aid ligands transportation between the active sites and surrounding solvent [27, 28], while the existence of access tunnels in xylanases has never been investigated yet. Using the CAVER algorithm [29], three tunnel-like structures in S7-xyl were found (Fig. 1a). Molecular docking analysis showed that the hydrolyzed xylopentaose was partially inserted inside the tun_1 (Fig. 1a). Thus, investigating the functionalities of these tunnel-like structures in S7-xyl would figure out whether they could be used as structure features for enzyme engineering in carbohydrate hydrolases.

As no prior knowledge was availability on how to rationally design the tunnel architecture in S7-xyl, we decided to reshape all three tunnels simultaneously to fully sample all possibilities. To avoid ruining tunnel architecture and minimize labor intensity, there residues V207, Q238, and W241 were selected as mutational targets based on the following principles: (i) lining all three tunnels (Additional file 1: Table S1), (ii) being not completely conserved in its homologous xylanases, (iii) being located at the loop regions. A combinatorial library with saturated mutations in the above three sites was constructed, resulting in a variant 254RL1 (V207N/Q238S/W241R) with a 340% increase in specific activity compared to S7-xyl (Fig. 1b).

### Deconvolution analysis

Deconvolution analysis was performed to dissect the effect of each substitution on the improved activity of 254RL1. As shown in Fig. 1b, eliminating the mutation of W241R (mutant S7-VQ: V207N/Q238S), V207N (mutant S7-QW: W241R/Q238S) and Q238S (mutant S7-VW: V207N/W241R) caused 23%, 50% and 52% loss in activity, respectively. The specific activities were further decreased in mutants with single residue substitution (Fig. 1b). These results demonstrated that all three mutations in 254RL1 contributed synergistically for the improved activity.

### Mechanism analysis for improved activity of the mutant 254RL1

**Fig. 2:**
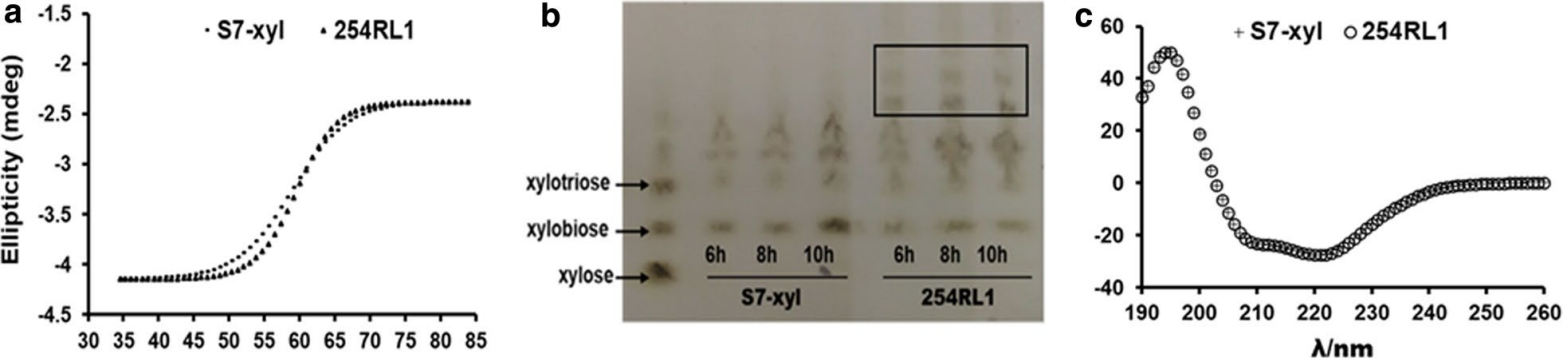
Biochemical characterization of wild-type S7-xyl and mutant 254RL1. **a** The thermal denaturation curves of S7-xyl and 254RL1. **b** TLC analysis of the hydrolysate of xylanases S7-xyl and 254RL1 on xylan. **c** The Far-UV (190–260 nm) CD spectra of S7-xyl and 254RL1

For enzymes with buried active sites, the catalytic cycle is composed of three major steps of substrates binding, enzyme catalysis, and products release [27, 30]. Kinetic analysis showed that the Michaelis constant (*K*_m_) of 254RL1 was slightly increased by 10%, while the catalytic efficiency (*k*_cat_/*K*_m_) of 254RL1 was increased by 350%, compared to wild-type S7-xyl (Table 1). The thermal denaturation curves showed that the melting temperatures (*T*_m_) of S7-xyl and 254RL1 are 58.8 ºC and 57.6 ºC, respectively (Fig. 2a). In addition, the catalytic residues (Glu159-Glu265) is kept unaltered in S7-xyl and 254RL1. Since the substrate binding and structural stability of S7-xyl and 254RL1 were not obviously changed, suggesting that the improved activity may attributed to accelerated products release in 254RL1. The above hypothesis was supported by the TLC results that larger end products were released from the reaction of 254RL1 (Fig. 2b).

**Table 1:** Kinetical parameters of S7-xyl and 254RL1

	*K*_m_ (mM)	*k*_cat_ (s^−1^)	*k*_cat_*/K*_m_ [(mM)^−1^ s^−1^]
Xyn10A	4.42 ± 0.12	4.57 ± 0.31	1.03
254RL1	4.04 ± 0.27	14.37 ± 0.21	3.54

To understand the structural variations that are responsible for improved activity of 254RL1, the far-UV CD spectra (190–260 nm) were used to assess protein folding and secondary structure contents of S7-xyl and 254RL1. As shown in Fig. 2c, there are no differences in secondary structure contents of S7-xyl and 254RL1. Then, we inspected the alterations of tunnel-like structures between S7-xyl and 254RL1 using CAVER analyst 2.0 [8], which revealed that the widths of tun_1 and tun_2 in 254RL1 were almost identical to S7-xyl, while their lengths were apparently reduced by 1.43 Å and 4.07 Å, respectively (Table 2). These changes were consistent with the observation that substitutions in 254RL1 reduced the steric hindrances near tunnels’ entrance (Fig. 1c).

**Table 2:** The tunnel parameters of wild-type S7-xyl and mutants

Xylanases	Avg BR (Å)			Avg L (Å)		
	tun_1	tun_2	tun_3	tun_1	tun_2	tun_3
S7-xyl	0.92	0.92	0.92	7.25	13.85	21.16
254RL1	0.95	0.95	0.91	5.82	9.78	21.63
238ML1	1.05	1.05	0.97	6.76	14.71	21.16

### Functional verification of tunnel-like structures

Since obvious alterations of tun_1 and tun_2 were observed in mutant 254RL1, we speculated that they may affect the enzyme activity. A good way to test the above speculation is to construct a mutant that has comparable tun_1 and tun_2 structures to that of wild-type S7-xyl. Thus, residues D163, W273, W325, R329, P208, and D246 lining tun_1 and/or tun_2 but not tun_3 of the second most active mutant S7-VQ (V207N/Q238S) were selected for in silico analysis, which found that introducing mutation W273 M would generate a mutant (286ML1: V207N/Q238S/W273 M) with similar tunnel parameters to S7-xyl (Table 2). As expected, the specific activity of 286ML1 was comparable to that of S7-xyl (Fig. 1b). Thus, the shorter tunnels promoted products release, resulting in improved activity of 254RL1.

Among these three tunnel-like structures, tun_3 has the longest length and its architecture was not obviously altered in mutants 254RL1 and 286ML1 (Table 2). To analyze whether tun_3 participated in products egress, residue T260 lining tun_3 was mutated to a bulky residue tryptophan to block tun_3 in S7-xyl. Enzymatic characterization showed that the specific activity of mutant T260W (Fig. 1b) was identical to S7-xyl, indicating that tun_3 is not related to the products release.

### Application assessment

**Fig. 3:**
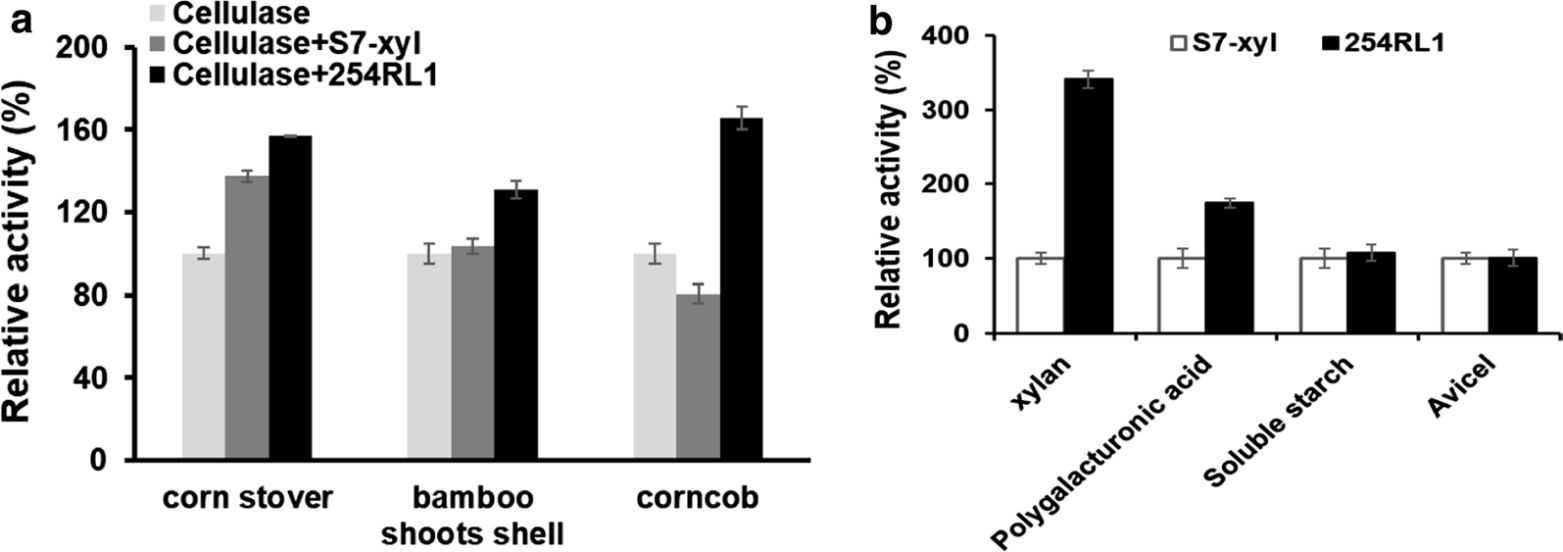
Application assessment of wild-type S7-xyl and mutant 254RL1. **a** The synergistic effects of wild-type S7-xyl and mutant 254RL1 in combination with cellulase Accellerase on different pre-treated biomass materials. **b** The substrate specificity of S7-xyl and 254RL1

Xylanase has been used as an important accessory enzyme in biorefinery. Application assessment showed that 254RL1 was superior to S7-xyl to enhance the hydrolysis efficiency of commercial cellulase Accellerase towards different pretreated lignocellulose materials, which yielded 67.8%, 14.3%, and 26.9% more xylose equivalents towards corncob [31], corn stover [32] and bamboo shoot shell [33], respectively (Fig. 3a). Specifically, there is no synergistic effect between wild-type S7-xyl and Accellerase against corncob. One possible explanation is that the S7-xyl activity was inhibited by the large amount of reducing sugars in pre-treated corncob. In contrast, significant improvement of corncob hydrolysis was observed when using the mutant 254RL1 as an accessory enzyme. Substrate specificity assay revealed that the hydrolytic activity towards another hemicellulose polygalacturonic acid was improved by 62% in 254RL1 compared to S7-xyl (Fig. 3b), which may be a contributor for the better application performance of 254RL1.

### General applicability of tunnel engineering in other GH10 xylanases

Since this is the first study of targeting tunnel-like structures for mutagenesis on xylanase, we attempted to clarify whether tunnel engineering could be a general strategy to optimize other xylanases’ properties. To keep the minimum time-consuming designing and labor-intensive screening, three criteria were used to identify xylanase candidates. Firstly, the candidates should have buried active sites while with different amino acid identities to S7-xyl. Secondly, the residues lining the tunnels should be highly conserved, so the mutations in 254RL1 could be directly applied to these enzymes. Finally, candidates should have been biochemically characterized to make sure they are bioactive xylanases.

**Fig. 4:**
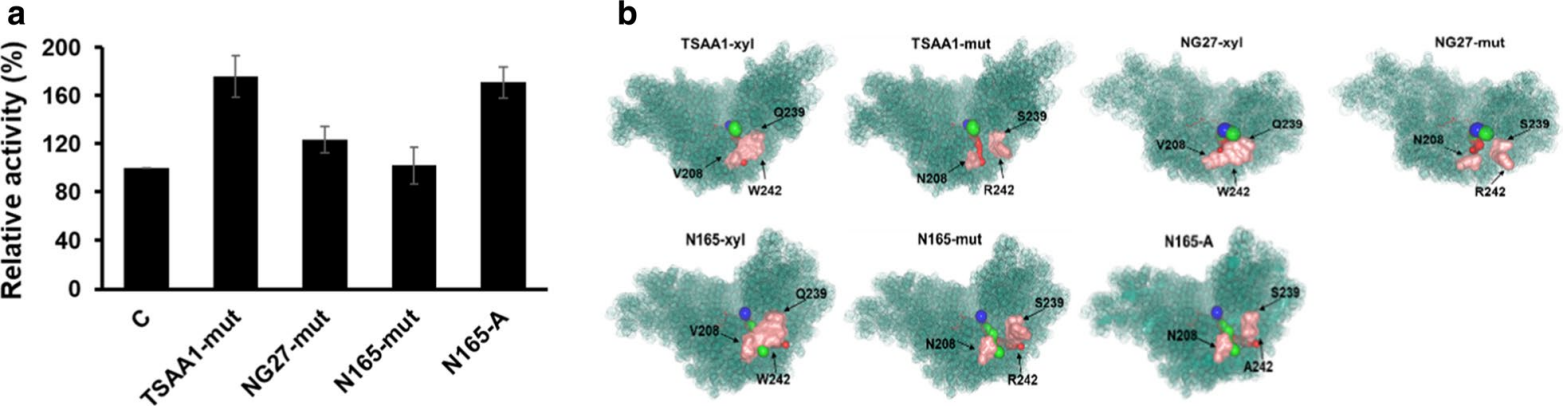
Application of tunnel engineering on other xylanases. **a** The relative activity of xylanase mutants. The activities of wild-type xylanases were set as control (C). **b** Structural comparison analysis of wild-type xylanases and the mutants. Backbone structures are shown as teal cartoon. Red sticks indicate catalytic residues. The mutated residues are shown as pink surface. The access tunnels were calculated by CAVER, named as tun_1 (blue), tun_2 (red) and tun_3 (green)

Three xylanases, NG27-xyl from *Bacillus* sp. strain NG-27 [24], TSAA1-xyl from *Geobacillus thermoleovorans* [25], and N165-xyl from *Bacillus* sp. N16-5 [26] sharing 87%, 69.9%, and 57.1% amino acid identity with S7-xyl, respectively, were selected for verifying our tunnel engineering strategy. The sites corresponding to V207, Q238 and W241 of S7-xyl were mutated to Asn, Ser, and Arg in the candidate xylanases, producing variants NG27-mut, TSAA1-mut and N165-mut with 80%, 20% and 7% increase in the specific activity, respectively (Fig. 4a). The visual structural variations around the access tunnels of wild-type xylanases and corresponding mutants are presented in Fig. 4b. Similar to 254RL1, the steric hindrance around tun_2 were obviously relieved in all mutants except for N165-mut (Fig. 4b). To verify whether reducing the steric hindrance on tun_2 of N165-xyl could improve its activity, R241 in N165-mut was mutated to a smaller residue alanine. The resultant variant N165-A exhibited a 170% enhancement in specific activity compared to N165-mut (Fig. 4a).

## Discussion

The access tunnels are important structure features that govern the catalytic cycle of enzymes with buried active sites, which existed in enzymes from all six enzyme classes [27]. In silico analysis showed that a twofold higher chance could be achieved to generate variants with improved properties by targeting the tunnel residues than other regions for mutagenesis [16]. Recently, tunnel engineering has been utilized to optimize the properties of enzymes acting on small substrates [34–36]. However, to date, this strategy has not been used to engineer enzymes catalyzing biopolymers. In this study, three tunnel-like structures were identified in xylanase S7-xyl using the CAVER algorithm. Instead of evaluating the effect of each tunnel-like structure on enzymatic properties, a combinatorial multi-sites saturation mutagenesis was performed to simultaneously reshape all three tunnels, which generated a mutant 254RL1 with a 340% increase in specific activity.

Some variations in mutants generated by multi-sites saturation mutagenesis are often redundant or ineffective [37], which would interfere the investigation of mechanisms underlying the changed enzymatic properties. Deconvolution of substitutions in 254RL1 (V207N/Q238S/W241R) revealed that all three mutations in 254RL1 contributed synergistically for the improved activity. Interestingly, substitution W241R decreased the specific activities of wild-type S7-xyl and variant Q238S, while it obviously improved the activity of variant S7-VQ (V207N/Q238S). The substitution W241R would be omitted in the iterative saturation mutagenesis, suggesting that combinatorial multi-sites saturation mutagenesis is superior to iterative saturation mutagenesis to sample the potential synergistic conformational and electrostatic effects of substitutions.

Biochemical characterization showed that there was no obvious alteration in structural stability and substrate affinity between S7-xyl and mutant 254RL1, while larger hydrolysates were released in the reaction of 254RL1. Inspection of the tunnel-like structures of S7-xyl and 254RL1 revealed that the lengths of tun_1 and tun_2 were apparently reduced by 1.43 Å and 4.07 Å in 254RL1, respectively. Because the access tunnels often involved in ligands transportation between the active sites and surrounding solvent [27, 28], previous studies have already demonstrated that mutation of the residues lining access tunnel would alter enzymes properties such as substrate specificity, enantioselectivity, products release [28, 35, 38]. It was speculated that the shorter tunnels in 254RL1 promoted products release and resulted in improved activity. In addition, the specific activities of three other xylanases were successfully improved by modifying their tunnel structures. These results clearly demonstrated that tunnel engineering is a powerful strategy to tailor the properties of biopolymer degrading enzymes, which can be used to engineer better biomass-degrading abilities in other lignocellulolytic enzymes with buried active sites.

## Conclusions

It is still controversial about the existence of access tunnels in enzymes degrading biopolymers like carbohydrate hydrolases. In this study, three tunnel-like structures in xylanase S7-xyl (PDB: 2UWF) were identified and simultaneously reshaped, resulting in a mutant 254RL1 with a 340% increase in specific activity compared to S7-xyl. Structure comparison and kinetic analysis indicated that these tunnel-like structures may function as products transportation pathways in S7-xyl. The mutant 254RL1 could act synergistically with cellulase against different pretreated biomass materials, making it an excellent candidate biocatalyst for biorefining application. In addition, the general application of tunnel engineering on carbohydrate hydrolases with buried active sites was verified in three other xylanases.

## Methods

### Chemicals, materials and gene synthesis

Restriction endonucleases and T_4_ DNA Ligase were purchased from New England Biolabs (NEB, USA), DNA polymerase was obtained from TakaRa (Dalian, China). The kits used for molecular cloning were purchased from Omega (USA). The codon-optimized xylanase genes and oligonucleotides were synthesized by Genecreate Co. Ltd (Wuhan, China). Beechwood xylan (Product No. X4252), soluble starch (Product No. V900508), polygalacturonic acid (Product No. 81325), and Avicel cellulose (Product No. 11363) were purchased from Sigma-Aldrich (USA). Beechwood xylan was used as substrate for enzymatic characterization. Soluble starch, polygalacturonic acid and Avicel cellulose were used for substrate specificity assays. 4-Nitrophenyl-β-xylobioside (Product code: O-PNPX2) was purchased from Megazyme (Ireland), which was used for kinetic analysis.

### Mutagenesis

To reduce codon redundancy and screening efforts, four primers (Additional file 1: Table S2) with two degeneracies (NDT, VMA) and two coding sequences (ATG, TGG) were used for site-saturation mutagenesis. As Q238 and W241 are adjacent, they were mutated within the same set of primer pairs. Primers were designed to have 15–25 bp overlapping sequences with one other. The PCR products were gel purified and mixed in equimolar ratio. Then, overlapping extension PCR was performed to assemble these fragments. The schematic view of library construction is shown in Additional file 1: Figure S1. For site-directed mutagenesis, primer pairs containing the appropriate base substitutions were designed and used to amplify the whole recombinant plasmids. The PCR products were digested by *Dpn* I and transformed into *E. coli* Rosetta (DE3). DNA sequences of all primers used to construct the saturated mutation library are listed in the Additional file 1: Table S2.

### Library screening

The transformants in the library were plated on LB agar plates containing 50 μg/mL kanamycin, 0.5% xylan and 0.5 mM IPTG. After cultured at 37 °C overnight, the colonies with halo diameter larger than the wild-type enzyme were picked into 96-well plates. The plates were incubated at 37 °C and 990×*g* for 12 h, then 0.5 mM IPTG was added to induce the expression of xylanases. After 3-h cultivation, cells were harvested by centrifugation, and resuspended in lysis buffer (50-mM Tris–HCl buffer, 150-mM NaCl, 1% Triton X-100, 50 mg/mL lysozyme, pH 7.4) at 37 °C for 2 h. After centrifugation, 10-μL supernatants were transferred into another 96-well plates containing 60-μL xylan solution. The enzymatic reaction was performed at 70 °C for 10 min, and stopped by adding 700-μL dinitrosalicylic acid (DNS) reagent, followed by boiling for 10 min and determination of the absorption at 540 nm. The mutants with higher enzymatic activities were purified to determine their specific activity as mentioned below.

### Expression and purification

Xylanase encoding genes from *B. halodurans* S7 (S7-xyl) (PDB: 2UWF), *Bacillus* sp. strain NG-27 (NG27-xyl) (PDB: 2FGL), *Geobacillus thermoleovorans* (TSAA1-xyl) (GenBank: AEW07375.1) and *Bacillus* sp. N16-5 (N165-xyl) (GenBank: ADI24221.1) and their mutants were cloned into plasmid pET-28a and expressed as N-terminal His-tagged proteins in *E. coli* Rosetta (DE3). Proteins were purified using a 5-ml HisTrap™ FF column (GE Healthcare) and desalted through a HiTrap™ Desalting column (GE Healthcare). The molecular weight and homogeneity of the purified proteins were evaluated by SDS-PAGE, and the protein concentration was determined by BCA Protein Assay Kit (Pierce) using bovine serum albumin as the standard.

### Enzyme assay and determination of kinetic parameters

The xylanase activity was evaluated by measuring the amount of reducing sugar released in the enzymatic hydrolysis by a modified DNS method [39]. The reaction mixtures containing 500-μL 1% (w/v) xylan in 20-mM glycine–NaOH buffer (pH 9.0) and appropriately diluted enzymes were incubated at 70 °C for 10 min, and stopped by adding 700-μL DNS reagent, followed by boiling for 10 min and determination of the absorption at 540 nm. To determine the substrate specificity, various carbohydrates, including soluble starch, avicel cellulose and polygalacturonic acid, at final concentration of 1% (w/v) were used as substrates. The hydrolysis reactions were conducted at 70 °C for 30 min, and the amount of released reducing sugars or galacturonic acid were measured by DNS method as described above. The Eadie–Hofstee plots were used to calculate kinetic parameters *K*_m_ and *V*_max_ according to the enzyme reactions [40]. All experiments to determine enzymatic properties were done at least in triplicate, and the error bars are standard deviations.

### Molecular modelling and tunnel exploration

The crystal structure of *B. halodurans* S7 xylanase (PDF: 2UWF) was obtained from the RSCB database. The tertiary structures of xylanases TSAA1-xyl, N165-xyl and all mutants were simulated using I-TASSER server [41]. Molecular docking between xylanase and ligand was performed using AutoDock software in YASARA [42], and the structure of hydrolyzed xylopentaose from crystal structure of xylanase XT6 (PDB: 3MMD) was used as ligand. To guide the docking to the active site of S7-xyl, a simulation cell was placed on 2UWF according to 3MMD. The top 25 structures rated by binding energy in YASARA were chosen for further analysis. The final docked conformation was selected manually with the ligand in PDB 3MMD as reference. CAVER Analyst 2.0 [29] was used to calculate transport tunnels in the wild-type xylanases and mutants using a probe radius of 0.9 Å, a shell radius of 3 Å, and a shell depth of 4 Å. The starting point for tunnel research was a point in the center of the active site cavity, which was defined by the geometric center of five atoms from surrounding residues (E159, N201, E265, H236, and D267, or equivalent residues in the mutants for S7-xyl).

### Thin-layer chromatography

Thin-layer chromatography (TLC) was firstly applied to detect the hydrolysis products of S7-xyl and the mutant 254RL1 on xylan. The reaction mixtures were spotted on TLC Silica gel 60F_10–20 cm_ (EMD/Merck, Darmstadt, Germany). and developed with a mixture of n-butanol, acetic acid and water (3:3:1, v/v/v). Spots were stained using 5% sulfuric acid and 95% phosphoric acid. Xylose (from Sinopharm Group, China), xylobiose, xylotriose, and xylotetraose (from Megazyme, Ireland) were used as standards.

### Circular dichroism spectroscopy

Circular dichroism (CD) spectra of S7-xyl and 254RL1 were collected between 190 and 260 nm with a 1-cm path-length quartz cuvette at a protein concentration of 0.1 mg/mL in glycine–NaOH buffer (pH 9.0, 20 mM). The spectropolarimeter and xenon lamp were warmed up for at least 30 min prior to experiments to minimize baseline signal drift. Ellipticity data were collected and a buffer blank was subtracted. The lengths and fractions of α-helixes and β-sheets were determined. The melting temperature (*T*_m_) of S7-xyl and 254RL1 was determined by monitoring ellipticity at 222 nm over the temperature range from 30 to 65 °C that gradually increased by 0.5 °C per minute.

### Enzymatic hydrolysis of pretreated lignocellulosic materials

To evaluate the hydrolysis efficiency of cellulase on different pre-treated lignocellulose materials when xylanase S7-xyl and 254RL1 were used as accessory enzyme, biomass including corncob treated by screw-steam-explosive extruder [31], corn stover obtained from sequential Fenton pretreatment and dilute NaOH extraction [32] and bamboo shoot shell treated by dilute alkalic salts sodium hypochlorite/sodium sulfide under the autoclave system [33], were used as substrates. The hydrolysis experiments were carried out at 55 °C in PBS buffer (pH 6.8) containing 1% (w/v) biomass, cellulase alone or in the combination with xylanase S7-xyl and 254RL1 were added to the reaction mixture. After 30-min incubation, the released reducing sugars were measured by DNS methods.

## Supplementary information

**Additional file 1: Table S1.** The residues lining each tunnel in 2UWF. **Table S2.** The sequences of primers used to construct the saturated mutant library in this study. **Figure S1.** Schematic illustration of the library construction.

## Data Availability

All data generated or analyzed during the study are included in this published article and its additional information files.

## Abbreviations

*E. coli*: *Escherichia coli*
GH: glycoside hydrolase
LB: Luria–Bertani medium
CD: circular dichroism
TLC: thin-layer chromatography
*K*_m_: Michaelis constant
*k*_cat_: turnover number
Tun_: tunnel
IPTG: isopropyl-β-d-thiogalactopyranoside
DNS: 3,5-dinitrosalicylic acid
PDB: protein database
SDS-PAGE: SDS-polyacrylamide gel electrophoresis
