# Supplementary material for: Tunnel engineering to accelerate product release for better biomass-degrading abilities in lignocellulolytic enzymes
Source: Biotechnol Biofuels. 2019 Nov 23;12:275. doi: 10.1186/s13068-019-1616-3 (PMC6874815; doi:10.1186/s13068-019-1616-3)

**Additional information**

**Tunnel engineering to accelerate product release for better biomass-degrading abilities in lignocellulolytic enzymes**

Zhenghui Lu^1^, Xinzhi Li^1^, Rui Zhang^1^, Li Yi^1^, Yanhe Ma^2^, Guimin Zhang^1^*

^1^State Key Laboratory of Biocatalysis and Enzyme Engineering, Hubei Collaborative Innovation Center for Green Transformation of Bio-resources, Hubei Key Laboratory of Industrial Biotechnology, School of Life Sciences, Hubei University, Wuhan, Hubei, China 430062

^2^Tianjin Institutes of Industrial Biotechnology, Chinese Academy of Science, Tianjin, 300308, China.

*To whom correspondence should be addressed. Tel: +86-27-88661746; Fax: +86-27-88663882; Email: zhangguimin@hubu.edu.cn, zhangguimin6@hotmail.com

**Table S1. The residues lining each tunnel in 2UWF.**

| Tunnel | Residues |
| --- | --- |
| tun_1 | D163, D202, Y203, N204, T205, E206, **V207**, H233, Q234, S235, H236, I237, **Q238**, **W241**, E265, W273, W325, R329 |
| tun_2 | D202, Y203, N204, T205, E206, **V207**, P208, H233, Q234, S235, H236, I237, **Q238**, **W241**, P242, S243, D246, E265 |
| tun_3 | D202, Y203, N204, T205, E206, **V207**, G232, H233, Q234, S235, H236, I237, **Q238**, I239, **W241**, P242, S243, I244, E245, T247, S250, F251, V263, E265, L266, R296, Q299, L300, L303, Y304 |

**Table S2. The sequences of primers used to construct the saturated mutant library in this study.**

| Primers | Sequences | Description |
| --- | --- | --- |
| F1 | cgggatccatgggtgtttttggtgagaaccaga | Amplify the 1# fragment of S7-xyl. |
| R1 | aaccaaattgtacaagtcatctctcttggatggAHNctcagtgttgtagtcgttgatgt |  |
|  | aaccaaattgtacaagtcatctctcttggatggTKBctcagtgttgtagtcgttgatgt |  |
|  | aaccaaattgtacaagtcatctctcttggatggCCActcagtgttgtagtcgttgatgt |  |
|  | aaccaaattgtacaagtcatctctcttggatggCATctcagtgttgtagtcgttgatgt |  |
| R2 | ccatcaattggaacaccctgctccaacaagtccttaaccaaattgtacaagtcatctct |  |
| R3 | aatgtgggattggtgaccaacaccatcaattggaacaccctgctc |  |
| F2 | ttggtcaccaatcccacattNDTattggtNNKccatccatcgaggacactagagctt | Amplify the 2# fragment of S7-xyl. |
|  | ttggtcaccaatcccacattVMAattggtNNKccatccatcgaggacactagagctt |  |
|  | ttggtcaccaatcccacattTGGattggtNNKccatccatcgaggacactagagctt |  |
|  | ttggtcaccaatcccacattATGattggtNNKccatccatcgaggacactagagctt |  |
| R4 | cccaagcttttaatcgatgattctccaataagctggc |  |

**Figure S1. Schematic illustration of the library construction**. The left fragment was firstly amplified by primer pairs of F1/R1, followed by F1/R2 and F1/R3. The right fragment was amplified by primer pairs of F2/R4. The left fragment and right fragment were assembled by overlapping PCR with primer pairs of F1/R4.


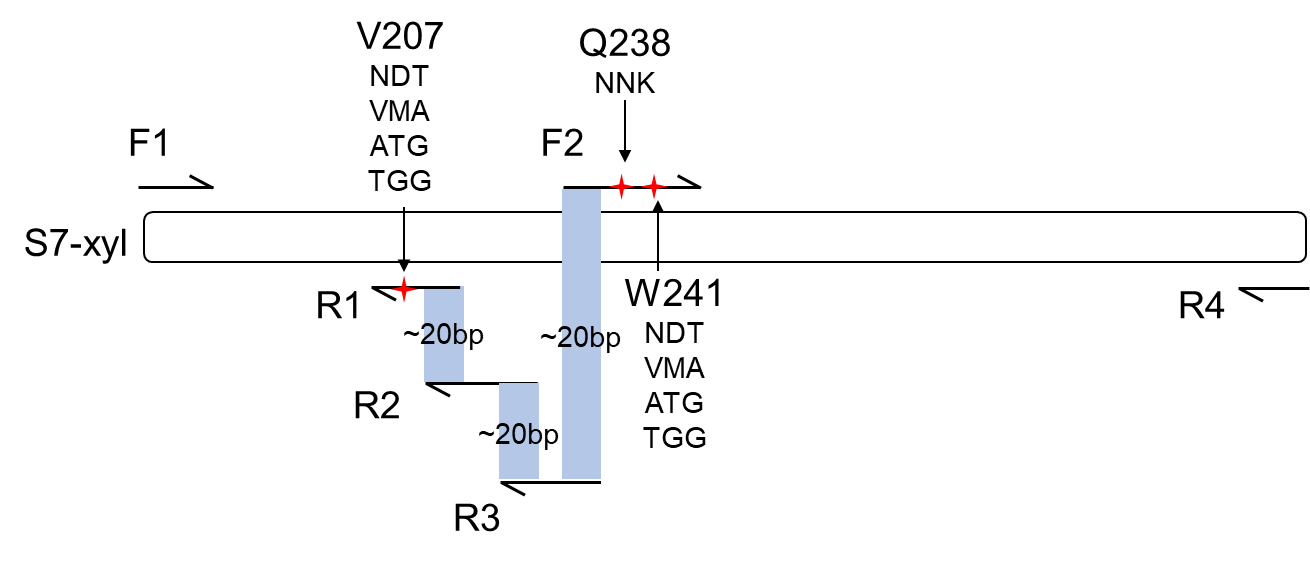

Supplement: Supplementary file 1 — Additional file 1: Table S1. The residues lining each tunnel in 2UWF. Table S2. The sequences of primers used to construct the saturated mutant library in this study. Figure S1. Schematic illustration of the library construction. [file 13068_2019_1616_MOESM1_ESM.docx]
